# Cardiocerebral hemodynamic characteristics of vasovagal syncope associated with cerebral autoregulation impairment

**DOI:** 10.3389/fneur.2026.1780645

**Published:** 2026-03-11

**Authors:** Meng Hou, Jingke Le, Lijie Ren, Liming Cao

**Affiliations:** 1 Guangzhou Medical University, Guangzhou, China; 2 Kuichong Community Health Service Center, Shenzhen Dapeng New District Medical and Health Group, Shenzhen, China; 3 Geriatric Hospital Affiliated with Wuhan University of Science and Technology, Wuhan, China; 4 Department of Neurology, The First Affiliated Hospital of Shenzhen University, Shenzhen, China; 5 Department of Neurology, Shenzhen Second People's Hospital, Shenzhen, China; 6 Hunan Provincial Key Laboratory of the Research and Development of Novel Pharmaceutical Preparations, Changsha Medical University, Changsha, China

**Keywords:** cardiocerebral hemodynamics, cerebral autoregulation, head-up tilt test, transcranial Doppler, vasovagal syncope

## Abstract

**Background:**

It is generally believed that cerebral hypoperfusion in patients with vasovagal syncope (VVS) is secondary to hypotension; however, current evidence suggests that this is not always the case. Thus, this study aimed to analyze the hemodynamic characteristics of patients with VVS and dynamic cerebral autoregulation (CA) dysfunction (referred to as CA-impaired VVS) to improve the diagnosis and treatment of this VVS subtype.

**Methods:**

This retrospective study included 143 patients with VVS who underwent the head-up tilt test (HUTT) using transcranial Doppler (TCD). Patients were divided into two groups based on pathogenesis: CA-impaired VVS and blood pressure drop-dominant VVS. Hemodynamic parameters, including systolic and diastolic blood pressure, heart rate, and cerebral blood flow velocity (CBFV), were compared between the two groups to further analyze the differences in cardiocerebral hemodynamic indices.

**Results:**

CA-impaired VVS accounted for 58% of the cases. During basic HUTT in the upright tilt position, the minimum systolic blood pressure (SBP), minimum diastolic blood pressure (DBP), and minimum mean arterial pressure (MAP) were significantly higher in the CA-impaired group than in the blood pressure drop-dominant group (*p* < 0.05). Similarly, during the sublingual nitroglycerin HUTT in the upright tilt position, the minimum SBP, DBP, and MAP were significantly higher in the CA-impaired group than in the blood pressure drop-dominant group (*p* < 0.05). After returning to the supine position, the mean SBP, DBP, and MAP remained significantly higher in the CA-impaired group than in the blood pressure drop-dominant group (*p* < 0.05). In addition, the positivity rates for orthostatic tachycardia in the CA-impaired and blood pressure drop-dominant VVS groups were 73.5 and 65.0%, respectively. The incidence of neurogenic orthostatic hypotension was 2.4% in the CA-impaired group, which was significantly lower than the 16.7% in the blood pressure drop-dominant group (*p* = 0.002).

**Conclusion:**

This study reveals the characteristics and differences in cardiocerebral hemodynamics between CA-impaired and blood pressure drop-dominant subtypes of VVS, deepens the understanding of VVS pathogenesis, facilitates the accurate diagnosis of VVS, and enhances the ability to interpret its results.

## 1 Introduction

Syncope is a common multidisciplinary problem in clinical settings. The lifetime incidence of syncope in children and adolescents ranges from approximately 15–25% (1). However, in adults, the lifetime incidence of syncope is approximately 40% (2, 3). Syncopal episodes are often accompanied by falls and secondary trauma, which may induce anxiety or depression in patients and significantly impair their quality of life and occupational capacity (4). Vasovagal syncope (VVS) is the most common subtype of syncope (5, 6). The pathogenesis of VVS stems from dysfunction of the autonomic nervous system, characterized by abnormally increased vagal tone and/or sudden reduction in sympathetic activity. This leads to abrupt peripheral vasodilation and/or significant bradycardia, which in turn causes a sharp drop in blood pressure, resulting in acute cerebral hypoperfusion and, ultimately, transient loss of consciousness (7). Other studies have also indicated that intracranial hypertension is an important mechanism in triggering orthostatic syncope (8). Under normal circumstances, the body relies on dynamic cerebral autoregulation (CA) to maintain stable cerebral perfusion. However, during the onset of VVS, even if dynamic CA is activated, its compensatory capacity may still be insufficient to counteract rapid hemodynamic changes, thereby triggering syncope. The head-up tilt test (HUTT) is recognized globally as the primary functional test for diagnosing VVS. It is widely regarded as the “gold standard” for clinical diagnosis, particularly for patients with suspected reflex syncope after excluding organic etiologies (9). Transcranial Doppler (TCD) continuously monitors cerebral blood flow velocity (CBFV) in the middle cerebral artery, as well as the pulsatility index (reflecting cerebrovascular resistance), during HUTT. A significant decrease in CBFV (up to 30–50%) is often observed, providing important functional evidence of cerebral hypoperfusion during VVS episodes (10, 11).

In clinical practice, we have observed that during HUTT combined with TCD monitoring, some patients with VVS exhibit a significant decrease in CBFV, accompanied by prodromal symptoms of syncope, even before the blood pressure drops (12–14). Studies have shown that TCD can detect a decrease in CBFV approximately 28 s before the reduction in mean arterial pressure (MAP) (15), suggesting that cerebrohemodynamic changes may occur early in the development of syncope. These findings indicate that cerebral hypoperfusion during prodromal symptoms and syncope is not necessarily secondary to systemic hypotension, but may involve CA dysfunction. It is generally believed that during a decrease in blood pressure, cerebral blood vessels undergo compensatory dilation through the CA mechanism to maintain cerebral perfusion and mitigate cerebral ischemia (16). However, in some patients with VVS, the CA mechanism may be impaired, leading to a significant decrease in CBFV, even preceding the onset of systemic hypotension. Monitoring with HUTT combined with TCD has revealed that when patients with VVS experience prodromal symptoms of syncope, the cerebral arteries do not undergo the expected compensatory dilation. Instead, they show signs of vasoconstriction, a phenomenon termed “paradoxical cerebral vasoconstriction.” This finding indicates CA dysfunction (10, 17). We hypothesized that CA dysfunction may play an important role in the pathogenesis of VVS. Based on this, patients in whom a significant decrease in CBFV occurs substantially earlier than the drop in blood pressure can be classified as “CA dysfunction-dominant VVS” (abbreviated as CA-impaired VVS), whereas those with synchronous decreases in CBFV and blood pressure are categorized as “blood pressure drop-dominant VVS.” Based on the combined detection technologies of HUTT and TCD, this study explored the hemodynamic characteristics of patients with CA-impaired VVS, thereby facilitating the development of more effective diagnostic, therapeutic, and preventive strategies.

## 2 Materials and methods

### 2.1 Study subjects

This retrospective study included 143 consecutive patients with VVS who visited the Second People’s Hospital of Shenzhen between August 2023 and December 2024 and completed HUTT combined with TCD, including 83 cases of CA dysfunction subtype VVS and 60 cases of blood pressure drop-dominant subtype VVS.

### 2.2 Definitions

In CA-impaired VVS, TCD monitoring shows a > 20% decrease in CBFV compared with baseline during syncope or presyncope. This decrease occurs before a significant drop in blood pressure, defined as systolic blood pressure (SBP) > 90 mmHg, diastolic blood pressure (DBP) > 50 mmHg, or MAP decrease <25% (calculated from individual baseline values).

In blood pressure drop-dominant VVS, CBFV decreases by >20% compared to the baseline during syncope or presyncope, but the timing of this decrease is not earlier than the onset of the significant blood pressure drop.

### 2.3 Inclusion and exclusion criteria, diagnosis, and positive judgment standards

The inclusion criteria were as follows: (1) age 7–80 years; (2) diagnosis of VVS based on detailed medical history collection, physical examination, and necessary auxiliary examinations; and (3) completion of HUTT combined with TCD, with test results meeting the positive diagnostic criteria for VVS.

Patients with any of the following conditions were excluded: severe intracranial or extracranial vascular stenosis (luminal diameter stenosis ≥70%), severe coronary artery stenosis (stenosis of major blood vessels ≥70%), severe aortic or mitral valve stenosis, severe hypertrophic obstructive cardiomyopathy, severe anemia (hemoglobin <60 g/L), severe arrhythmias (e.g., high-grade atrioventricular block and sustained ventricular tachycardia), severe hypertension (SBP > 180 mmHg and/or DBP > 110 mmHg), inability to cooperate during the examination owing to severe mental illness or cognitive impairment, and pregnancy.

The diagnosis of VVS was mainly based on a typical medical history characterized by sudden, transient, and self-limited loss of consciousness induced by specific triggers. Relevant auxiliary examinations were also considered, including electrocardiography (ECG), echocardiography (to rule out structural heart disease), electroencephalography (EEG), or head computed tomography/magnetic resonance imaging, when necessary (to rule out central nervous system diseases), and HUTT for auxiliary diagnosis. The final diagnosis was confirmed after excluding etiologies such as epilepsy, hypoglycemia or other metabolic abnormalities, cardiogenic syncope, drug overdose, alcohol intoxication, head trauma, psychogenic pseudosyncope, and other identifiable causes of disturbance of consciousness.

The criteria for interpreting positive HUTT results (9, 18) were as follows: (1) definite prodromal symptoms of syncope (such as amaurosis fugax, significant dizziness, and pale complexion) or syncope occurring during the test; (2) occurrence of any of the following hemodynamic changes during basic HUTT (BHUTT) or sublingual nitroglycerin HUT (SNHUTT): SBP < 80 mmHg and DBP < 50 mmHg; MAP decrease by >25%; and SBP < 90 mmHg accompanied by prodromal symptoms of syncope; (3) ECG showing any of the following rhythm changes: sinus bradycardia <60 beats per minute (in children and adolescents), <40 beats per minute (in adults aged ≥18 years); sinus arrest >3 s; heart rate (HR) decrease by >20%; transient second-degree or higher atrioventricular block or junctional rhythm.

### 2.4 Study methods

Clinical data of patients (including sex, age, body mass index [BMI], medical history, and medications potentially influencing autonomic function), as well as data on symptoms, HR, blood pressure, and changes in CBFV during the combined HUTT and TCD, were collected retrospectively. This study was approved by the Ethics Committee of Shenzhen Second People’s Hospital (approval number: 2023–088-02PJ).

### 2.5 Preparation and operating procedures for HUTT (18)

(1) Patient Preparation: Patients were required to fast for at least 4 h before the examination and avoid consuming beverages containing excitatory substances such as coffee and tea. They were asked to empty their bladders before the test.

(2) Test Environment and Safety Protection: The room was kept quiet with gentle light, the room temperature was controlled at 20–26 °C to avoid autonomic nervous reactions induced by excessive cold or heat, and patients rested in a supine position for 5–10 min and were properly secured with a thoracoabdominal belt. The belt was adjusted to a comfortable level to prevent the patient from falling out of the bed or from sustaining injury during postural changes.

(3) Evaluation of Patients with Syncope: Before performing HUTT, a comprehensive evaluation was conducted to identify contraindications. This evaluation involved a detailed medical history, a systematic physical examination, and results of necessary auxiliary examinations (such as echocardiography, routine ECG, EEG, and/or 24-h ambulatory ECG) to ensure patient safety during the examination.

(4) Test Protocol: The HUTT 822-A Head-Up Tilt Test System (Juchi Medical Co., Ltd., Beijing, China) was used. First, a BHUTT was performed: Patients rested in a supine position for 5–10 min, and baseline blood pressure, HR, ECG, and CBFV were recorded. Subsequently, the bed was quickly elevated to a 70° head-up tilt position within 8 s and maintained for 20–45 min. During this period, if the patient experienced syncope or intolerable prodromal symptoms of syncope, the test was terminated immediately, and the patient was rapidly returned to the supine position (<8 s). If the BHUTT result was negative, SNHUTT was performed. While maintaining the 70° tilt position, patients received sublingual nitroglycerin at a dose of 4–6 μg/kg (maximum dose ≤300 μg), with a standardized adult dose of 0.3 mg. After administration, the upright tilt position was maintained for an additional 15 min of observation until syncope or intolerable prodromal symptoms occurred. Once such symptoms appeared, the test was stopped immediately, and the patient was quickly returned to the supine position. Throughout the examination, changes in the patient’s symptoms, blood pressure, HR, ECG, and CBFV were continuously recorded at each stage: baseline → tilt position → drug provocation → return to the supine position.

### 2.6 Methods for synchronous TCD cerebral blood flow monitoring (11)

**Figure 1:**
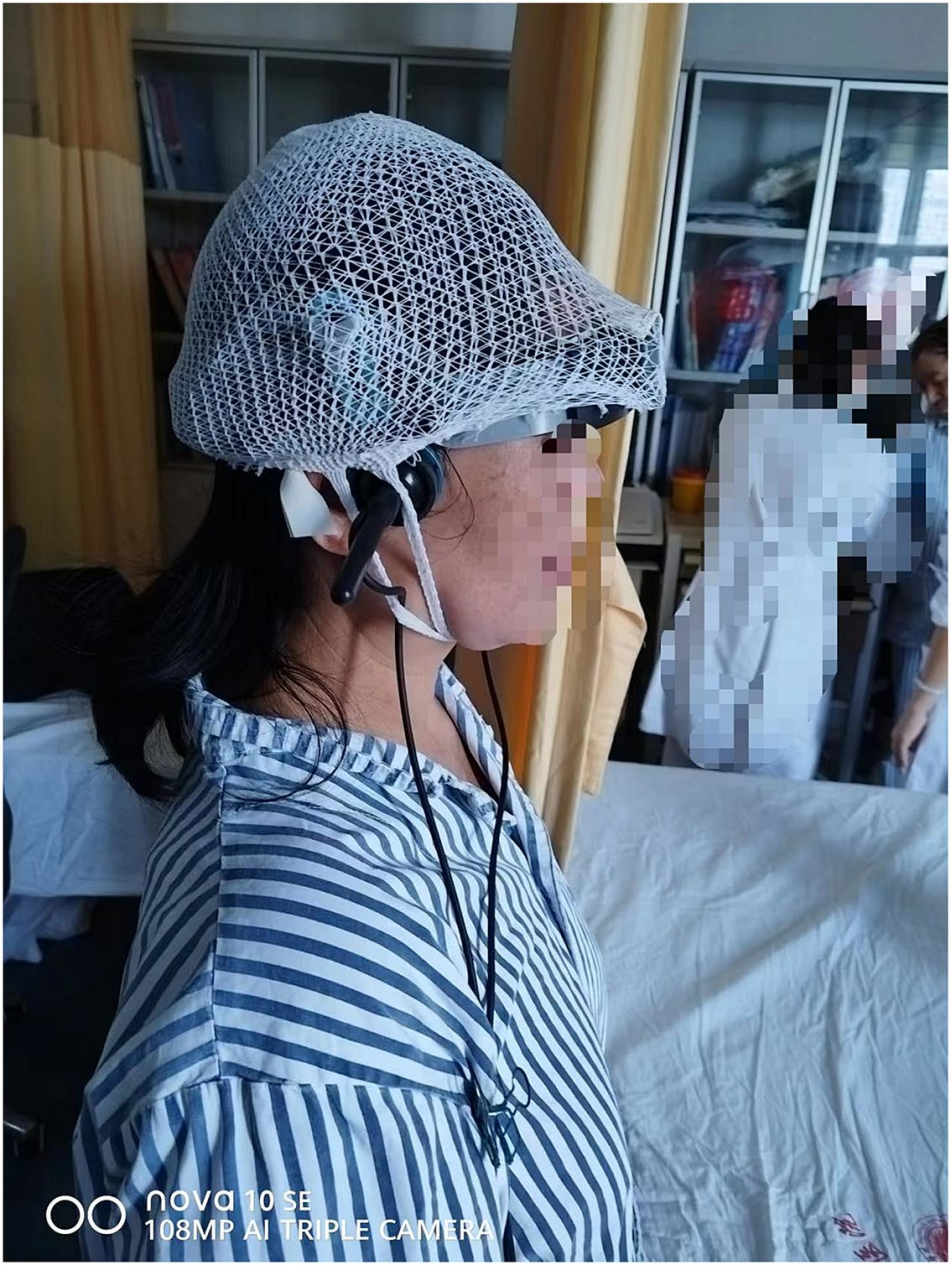
Fixation of the transcranial Doppler (TCD) head frame and probe with a multilayer elastic mesh cap. After confirming the probe position, the multilayer elastic mesh cap is worn on the subject, covering the forehead, probes, and occipital frame of the subject’s head, whereas the handle remains uncovered.

During the HUTT, EMS-9D Type TCD (Delica Medical Equipment Co., Ltd., Shenzhen, China) was used to continuously monitor cerebral blood flow. A 2.0 MHz probe was used to monitor the CBFV of the unilateral or bilateral middle cerebral arteries at a depth of 50–60 mm through the bilateral temporal windows, and the changes in end-systolic, end-diastolic, mean CBFV, and vascular resistance index were recorded. To reduce the TCD probe displacement caused by postural changes (supine position → tilt position → supine position) and ensure the continuous acquisition of valid signals, a multilayer elastic mesh cap independently developed by our team with independent intellectual property rights (Patent No.: ZL 202420167645.3; Figure 1) was used to firmly fix the TCD head frame and probe. This ensured stable and continuous cerebral hemodynamic monitoring with the TCD probe throughout the entire HUTT.

### 2.7 Statistical methods

Statistical analysis was performed using SPSS 26.0 software. Categorical data are expressed as rates and constituent ratios, and comparisons are presented in frequency tables. The chi-square test for contingency tables or Fisher’s exact test was selected based on data characteristics. Normally distributed continuous data are expressed as mean ± standard deviation (*x̄* ± *s*), and comparisons between two groups were performed using the independent samples *t*-test. Non-normally distributed continuous data are reported as median and interquartile range [P50 (P25, P75)], and the nonparametric Mann–Whitney *U* test was used for between-group comparisons. All statistical inferences were based on two-tailed tests, with a *p*-value < 0.05 considered statistically significant.

## 3 Study results

In this study, 83 patients (58%) were in the CA-impaired VVS group, and 60 (42%) were in the blood pressure drop-dominant VVS group.

Baseline demographic and clinical characteristics were comparable between the two groups (Table 1). There were no significant differences in sex, age, BMI, Comorbidities, or Medications known to profoundly affect autonomic reflexes between the two groups (*p* > 0.05).

**Table 1:** Comparison of baseline clinical characteristics between the two groups.

Variable	CA-impaired VVS (*n* = 83)	Blood pressure drop-dominant VVS (*n* = 60)	*t*/*χ*^2^/*Z*	*P*-value
Age, years	36.0 (26.0, 46.0)	41.0 (24.3, 52.0)	Z = −0.663	0.507
Sex, female, *n* (%)	43 (51.8%)	34 (56.7%)	*χ*^2^ = 0.331	0.565
Body mass index, kg/m^2^	22.5 ± 3.30	22.3 ± 3.88	*t* = −0.426	0.671
Comorbidities				
Hypertension, *n* (%)	9 (10.8%)	9 (15.0%)	*χ*^2^ = 0.547	0.460
Diabetes mellitus, *n* (%)	2 (2.4%)	6 (10.0%)	*χ*^2^ = 3.799	0.069
Stroke, *n* (%)	3 (3.6%)	6 (10.0%)	*χ*^2^ = 2.408	0.166
Anxiety/Depression, *n* (%)	5 (6.0%)	2 (3.3%)	*χ*^2^ = 0.542	0.699
Medications				
β-blockers, *n* (%)	2(2.4%)	5(8.3%)	*χ*^2^ = 2.625	0.131
SSRIs/SNRIs, *n* (%)	5(6.0%)	2(3.3%)	*χ*^2^ = 0.542	0.699
Midodrine or other α-agonists, *n* (%)	0(0.0%)	0(0.0%)		
Diuretics, *n* (%)	0(0.0%)	1(1.7%)	*χ*^2^ = 1.393	0.420
Anticholinergics, *n* (%)	1(1.2%)	0(0.0%)	*χ*^2^ = 0.728	1.000

SSRIs, selective serotonin reuptake inhibitors; SNRIs, serotonin-norepinephrine reuptake inhibitors.

### 3.1 HUTT results

The positive rates of single HUTT results in the CA-impaired and blood pressure drop-dominant VVS groups were 18.1% (15/83) and 100% (60/60), respectively (*p* < 0.001, Table 2). Here, “single HUTT positivity” refers specifically to conventional diagnostic positivity determined solely by hemodynamic criteria (blood pressure, HR, and ECG changes) observed during either the BHUTT or the SNHUTT, without incorporation of TCD data.

**Table 2:** Comparison of HUTT and TCD results between patients with CA-impaired VVS and blood pressure drop-dominant VVS.

Variable		CA-impaired VVS (*n* = 83)	Blood pressure drop-dominant VVS (*n* = 60)	*χ*^2^	*P*
HUTT of results	Negative	68 (81.9%)	0 (0.0%)	93.725	<0.001
	Positive	15 (18.1%)	60 (100.0%)		
Induction of presyncope	Yes	70 (84.3%)	56 (93.3%)	2.690	0.101
	No	13 (15.7%)	4 (6.7%)		

In the HUTT, there was no statistically significant difference in the induction of prodromal symptoms of syncope between the CA-impaired and blood pressure drop-dominant VVS groups (Table 2).

### 3.2 Comparison of cardiocerebral hemodynamics between the two groups

Regarding combined HUTT and TCD, at baseline, there were no statistically significant differences in mean SBP, mean DBP, MAP, mean HR, or mean CBFV between patients with CA-impaired VVS and those with blood pressure drop-dominant VVS (all *p* > 0.05).

In the 70° BHUTT position, the minimum SBP, DBP, and MAP were significantly higher in the CA-impaired group than in the blood pressure drop-dominant group (all *p* < 0.05).

In the 70° SNHUTT position, the minimum SBP, DBP, and MAP were significantly higher in the CA-impaired group than in the blood pressure drop-dominant group (all *p* < 0.05).

In the 0° supine position, the mean SBP, mean DBP, and MAP were significantly higher in the CA-impaired group than in the blood pressure drop-dominant group (all *p* < 0.05) (Table 3).

**Table 3:** Comparison of cardiocerebral hemodynamic parameters between patients with CA-impaired and blood pressure drop-dominant VVS.

Variable		CA-impaired VVS (*n* = 83)	Blood pressure drop-dominant VVS (*n* = 60)	*χ*^2^/*t*/*Z*	*P*
Baseline in the supine position	SBP, mmHg	117.0 (108.0, 128.0)	114.0 (105.0, 126.0)	*Z* = −0.917	0.359
	DBP, mmHg	74.5 (70.0, 85.3)	74.0 (66.3, 81.0)	*Z* = −1.343	0.179
	MAP, mmHg	88.0 (82.0, 99.0)	87.0 (78.3, 97.0)	*Z* = −1.214	0.225
	HR, bpm	72.3 ± 10.6	72.3 ± 11.3	*t* = −0.036	0.971
	CBFV, cm/s	55.0 (50.0, 65.0)	60.0 (50.0, 64.8)	*Z* = −0.346	0.729
Baseline head-up tilt position	Min SBP, mmHg	110.0 (105.0, 120.0)	107.0 (92.3, 117.0)	*Z* = −2.192	0.028
	Min DBP, mmHg	75.5 ± 10.4	71.1 ± 10.2	*t* = 2.530	0.012
	Max HR, bpm	91.0 (81.0, 102)	88.0 (80.3, 103.0)	*Z* = −0.401	0.688
	Min MAP, mmHg	86.0 (81.0, 97.0)	83.5 (76.3, 90.8)	*Z* = −2.768	0.006
Tilted position during drug administration	Min SBP, mmHg	109.0 ± 11.9	93.3 ± 14.2	*t* = 6.590	<0.001
	Min DBP, mmHg	72.1 ± 10.1	59.9 ± 11.0	*t* = 6.535	<0.001
	Max HR, bpm	109.0 ± 17.2	111.0 ± 20.2	*t* = −0.685	0.495
	Min MAP, mmHg	85.0 ± 10.6	71.3 ± 11.8	*t* = 6.856	<0.001
	Minimum CBFV, cm/s	35.0 (30.0, 40.0)	30.0 (30.0, 40.0)	*Z* = −0.741	0.459
Returned to the supine position	SBP, mmHg	113.0 (106.0, 121.0)	106.0 (97.3, 115.0)	*Z* = −3.757	<0.001
	DBP, mmHg	73.1 ± 9.9	65.7 ± 9.9	*t* = −4.404	<0.001
	MAP, mmHg	86.8 ± 9.9	79.4 ± 9.8	*t* = −4.377	<0.001
	HR, bpm	77.4 ± 13.0	74.1 ± 12.6	*t* = −1.495	0.137
	CBFV returned to baseline (±10%)	71.0 (85.5%)	49.0 (81.7%)	*χ*^2^ = 0.388	0.534

CBFV, cerebral blood flow velocity; HR, heart rate.

### 3.3 Postural tachycardia and orthostatic hypotension findings

Three key findings were noted regarding HUTT. First, the positive rates of orthostatic postural tachycardia in the CA-impaired and blood pressure drop-dominant VVS groups were 73.5 and 65.0%, respectively (*p* > 0.05). Second, in the CA-impaired VVS group, the positive rates of postural tachycardia during BHUTT and SNHUTT were 22.9 and 50.6%, respectively (*p* > 0.05). In the blood pressure drop-dominant VVS group, the positive rates of postural tachycardia during BHUTT and SNHUTT were 21.7 and 43.3%, respectively (*p* > 0.05). Third, the distribution of orthostatic hypotension (OH) incidence among the various subtypes in the CA-impaired and blood pressure drop-dominant VVS groups is shown in Table 4.

**Table 4:** Intergroup comparison of postural tachycardia and orthostatic hypotension between patients with CA-impaired and blood pressure drop-dominant VVS.

Variable		CA-impaired VVS (*n* = 83)	blood pressure drop-dominant VVS (*n* = 60)	*χ*^2^	*P*
Postural tachycardia	Baseline	19 (22.9%)	13 (21.7%)	*χ*^2^ = 0.030	0.862
	Drug-induced status	42 (50.6%)	26 (43.3%)	*χ*^2^ = 0.738	0.390
OH	Classic OH	2 (2.4%)	6 (10.0%)	*F* = 3.799	0.069
	Delayed OH	4 (4.8%)	8 (13.3%)	*χ*^2^ = 3.284	0.070
	Non-neurogenic OH	4 (4.8%)	4 (6.7%)	*F* = 0.225	0.720
	Neurogenic OH	2 (2.4%)	10 (16.7%)	*χ*^2^ = 9.208	0.002

OH, orthostatic hypotension.

## 4 Discussion

This study found that CA-impaired VVS is not uncommon. The combination of HUTT and TCD monitoring exhibited significant value in identifying patients with CA-impaired VVS, as it can effectively prevent missed diagnoses that could occur when HUTT alone is used. This combined protocol is particularly suitable for evaluating VVS dominated by CA impairment, significantly improving the sensitivity and safety of diagnosis.

### 4.1 Pathophysiological mechanisms of VVS caused by CA disturbance

Studies have shown that CA disturbance may be an important mechanism underlying early decreases in CBFV in patients with VVS, and this phenomenon involves complex pathophysiological processes. CA maintains cerebral perfusion stability mainly through the following mechanisms. (1) Myogenic response: Through the response of cerebral arteriolar smooth muscle to changes in transmural pressure, CA actively regulates vascular diameter (resistance) to maintain relative constancy of cerebral blood flow during blood pressure fluctuations (14); (2) Metabolic regulation: Changes in the local chemical environment of brain tissue (such as partial pressures of CO₂, O₂, and pH value) can directly affect cerebral vascular tone, among which CO₂ concentration is the most critical physiological factor regulating cerebral blood flow (19, 20); (3) Autonomic nervous regulation: Sympathetic (adrenergic) and parasympathetic (cholinergic) nerve fibers innervate larger cerebral arteries, which can regulate the contraction and relaxation of cerebral blood vessels under stress or specific pathological conditions (21, 22); (4) Endothelial cell regulation: Vascular endothelial cells release various active substances through paracrine mode, such as vasodilatory nitric oxide, vasoconstrictive endothelin-1, and thromboxane A₂, to dynamically regulate cerebral vascular tone (23). These mechanisms do not operate in isolation, but are interrelated and synergistic, jointly ensuring the stability of cerebral blood flow under different physiological and pathological conditions.

### 4.2 Hemodynamic differences between patients with CA-impaired and blood pressure drop-dominant VVS

(1) In the combined HUTT and TCD monitoring, during the tilted position of BHUTT and SNHUTT, as well as when patients returned to the supine position after HUTT completion, the minimum SBP, DBP, and MAP in the CA-impaired VVS group were significantly higher than those in the blood pressure drop-dominant VVS group. This suggests that cerebral hypoperfusion and related symptoms (such as presyncope) in patients with CA-impaired VVS do not require significant systemic hypotension; their symptom initiation threshold is lower, and they exhibit a strong compensatory capacity for blood pressure regulation. In contrast, blood pressure drop-dominant VVS may be closely associated with impaired blood pressure regulation. This study showed that patients with CA-impaired VVS were less likely to have blood pressure drops, meeting the positive diagnostic criteria for HUTT (e.g., SBP ≤ 80 mmHg), but were more prone to false-negative HUTT results. Patients with impaired CA cannot rapidly and effectively maintain cerebral perfusion stability under stress, such as postural changes, indicating that cerebral hypoperfusion and syncope are not always secondary to an obvious blood pressure drop but may arise from early decompensation of the CA mechanism.

(2) This study found that the number of patients meeting the criteria for neurogenic OH was significantly lower in the CA-impaired VVS group than in the blood pressure drop-dominant VVS group. This result is consistent with the aforementioned analysis, namely that blood pressure drop-dominant VVS is more likely to be associated with impaired compensatory autonomic nervous system function under acute postural stress. Under normal circumstances, orthostatic tilt can induce a compensatory increase in HR through the baroreflex, typically by 10–15 beats per minute. In neurogenic OH, however, patients exhibit an SBP decrease ≥20 mmHg or a DBP decrease ≥10 mmHg in the upright position, accompanied by an HR increase <10 beats per minute, indicating impaired sympathetically mediated vasoconstriction and HR acceleration responses (18, 24). In patients with VVS, dysfunction or impairment of the sympathetic and vagus nerves is common, such that when blood pressure drops, HR cannot promptly increase to enhance cardiac output (25). This study suggests that patients with blood pressure drop-dominant VVS are more prone to inadequate compensation of cardiovascular autonomic reflex function than those with CA-impaired VVS, reflecting more significant dysfunction in their blood pressure regulatory homeostasis.

(3) This study found that the minimum SBP, DBP, and MAP were significantly higher in the CA-impaired VVS group than in the blood pressure drop-dominant VVS group in the tilted position. This phenomenon suggests that patients with CA disturbance often experience a sharp decrease in CBFV due to impaired CA function before a significant blood pressure reduction occurs, thereby triggering presyncope or loss of consciousness. In contrast, blood pressure drop-dominant VVS relies mainly on obvious hypotensive events as the driving factors for syncope. In addition, after HUTT, when patients returned to the supine position, blood pressure recovery remained significantly higher in the CA-impaired VVS group than in the blood pressure drop-dominant VVS group. This result may be related to the latter experiencing more significant excessive vagal activation and sympathetic inhibition, leading to transient inhibition of the cardiovascular system and delayed blood pressure recovery. However, the former, despite showing cerebral hypoperfusion, had less systemic circulatory disturbance, resulting in faster blood pressure rebound. Therefore, “higher blood pressure” or “faster recovery” should not be interpreted simply as “stronger autonomic nervous function” but rather understood hierarchically in the context of cerebral hemodynamics.

### 4.3 Hemodynamic test in patients with VVS of CA impairment

The HUTT combined with TCD can more comprehensively evaluate the cardiopulmonary and cerebral hemodynamic status of patients with VVS and is particularly helpful for identifying VVS of the CA impairment type.

The HUTT continuously monitors patients’ symptoms, blood pressure, HR, and ECG changes at different stages (baseline supine position – tilt phase – drug provocation [if necessary] – recovery to the supine position), and analyzes and dynamically generates visualizable data to assist interpretation. In this study, among patients with CA-impaired VVS who underwent HUTT alone without a TCD combination, only 15 (18.1%) responded positively. Studies have demonstrated that although HUTT has high specificity for diagnosing VVS, its sensitivity is relatively low (26). In contrast, the sensitivity of SNHUTT can reach 77%, with a specificity as high as 93% (27, 28). The results of this study indicate that, when relying solely on BHUTT and SNHUTT, a considerable number of patients with CA-impaired VVS will yield false-negative results. Notably, in patients with VVS, blood pressure and HR do not decrease significantly during prodromal syncope or syncopal episodes, suggesting that the reduction in CBFV is not entirely secondary to decreased blood pressure or cardiac output, but rather stems from CA dysfunction, which poses an important challenge to the traditional mechanism of VVS (29).

Since the bilateral middle cerebral arteries supply approximately 60–70% of the total cerebral blood flow, TCD is commonly used clinically to monitor CBFV in these arteries as a representative indicator of global cerebral perfusion (16). The results of this study show that a considerable proportion of patients exhibit a significant decrease in CBFV before symptom onset, which occurs earlier than the obvious reduction in arterial blood pressure. In the traditional diagnostic process for VVS, HUTT mainly relies on changes in blood pressure and HR as positive criteria. However, patients with CA-impaired VVS often present with cerebral blood flow regulation failure rather than circulatory depression. Thus, even with a relatively stable blood pressure, severe cerebral hypoperfusion may still occur, increasing the risk of a missed diagnosis. This suggests that examiners need to simultaneously monitor dynamic changes in CBFV during HUTT, especially for patients with cerebral hypoperfusion symptoms such as dizziness and amaurosis fugax, but without significant blood pressure fluctuations. This study highlights the value of TCD combined with HUTT; by providing real-time early warning of a deteriorating trend in CBFV, it not only improves diagnostic sensitivity for CA-impaired VVS but also prevents adverse events induced by excessive cerebral hypoperfusion, enhancing the safety of the test. In the tilted upright position, if patients have impaired or even decompensated CA, it will lead to a significant decrease in CBFV, making it impossible to maintain a steady state of cerebral perfusion (14, 30). TCD combined with HUTT enables simultaneous assessment of cardiopulmonary and cerebral hemodynamics, facilitating the early identification of VVS subtypes characterized by CA dysfunction, which is clinically significant for precise subtyping and individualized management (31).

### 4.4 Management of VVS with CA impairment

Currently, research on specific prophylaxis and treatment for CA-impaired VVS is limited. Given that it is a subtype of VVS, clinical practice can refer to comprehensive management strategies for VVS, which mainly include the following aspects:

(1) Identification and Avoidance of Triggers: Priority should be given to populations with low BMI (32, 33), a history of transient loss of consciousness, or recurrent orthostatic dizziness. Patients and their family members should be guided to systematically document the circumstances, prodromal symptoms, and behavioral patterns prior to each episode to identify and avoid individualized triggers.

(2) Lifestyle Modifications: (A) Avoidance of prolonged maintenance of a single posture: Prolonged sitting or standing can lead to lower extremity venous stasis and reduced venous return, thereby increasing the risk of syncope. Patients should be advised to stand up and engage in light activity for 3–5 min every 30–60 min to promote the lower limb muscle pump effect and maintain circulatory stability. (B) Moderate physical exercise: Regular low-to-moderate-intensity aerobic exercises (e.g., brisk walking, cycling, and swimming) can improve autonomic nervous regulation and cardiovascular adaptability (34). The intensity of the exercise should be reduced gradually, rather than abruptly, at the end of the workout to avoid inducing exercise-related syncope. (C) Dietary and work-rest schedule management: Regular meals should be maintained, and overeating or prolonged fasting should be avoided. In particular, consuming alcohol without eating should be avoided. For patients with recurrent episodes who have no contraindications such as hypertension or heart failure, an appropriate increase in fluid and salt intake is recommended to expand the effective circulating blood volume (5). Moreover, adequate sleep should be ensured, with avoidance of staying up late and overfatigue, to maintain balance in autonomic nervous function.

(3) Psychological Stress Management: Active psychological stress management helps improve symptom control in patients with VVS (35). Specifically, psychological stress can be alleviated through physical exercise, social support, psychological counseling, or cognitive behavioral therapy, thereby regulating autonomic nervous system dysfunction.

(4) Physical Counterpressure Maneuvers (PCM) and Tilt Training: PCMs are indicated for patients with definite prodromal syncope symptoms. Patients should be instructed to immediately perform PCMs (e.g., leg crossing, muscle tensing, and fist clenching with elbow flexion against resistance) at the onset of prodromal symptoms. These maneuvers can prevent syncope by increasing peripheral vascular resistance and venous return (36). Tilt training: Progressive upright posture training can gradually improve baroreceptor sensitivity and enhance orthostatic tolerance (5).

(5) Pharmacological Therapy: (A) Alpha-adrenergic agonists (e.g., midodrine) (37) are commonly used in patients with OH. By constricting peripheral blood vessels and increasing peripheral vascular resistance, these agents can improve blood pressure and cerebral blood flow perfusion. (B) Selective serotonin reuptake inhibitors can be administered to patients with VVS and anxiety or depression. They may reduce syncopal episodes by regulating central autonomic nervous function, but relevant evidence is limited; therefore, they are not recommended as first-line therapy (38).

### 4.5 Advantages and limitations of this study

This study systematically describes the cardiopulmonary and cerebral hemodynamic characteristics of “CA-impaired VVS,” focusing on the dynamic changes in CBFV, blood pressure, and HR. For patients with clinically suspected VVS but negative conventional HUTT results, especially those complaining of “dizziness” but with stable blood pressure, combined HUTT and TCD may be considered to evaluate for hemodynamic abnormalities related to CA impairment.

This was a single-center retrospective study, and only patients who successfully completed the combined HUTT and TCD were included, potentially introducing selection bias. Although this study revealed hemodynamic differences between CA-impaired VVS and blood pressure drop-dominant VVS, further research is needed to explore the dynamic relationships among hemodynamic indicators during HUTT.

In the future, prospective, multicenter, large-sample clinical studies should be conducted to systematically compare the differences in clinical manifestations, hemodynamic evolution, and long-term prognosis between “CA-impaired VVS” and “blood pressure drop-dominant VVS,” so as to verify the rationality of its status as an independent subtype and gradually establish diagnostic criteria. Future studies can use artificial intelligence to analyze the dynamic correlations among CBFV, blood pressure, and HR in a time series. Artificial intelligence can also be used to integrate dynamic TCD monitoring data and HUTT results to construct individualized prediction models, which can be applied to assess the risk of onset and response to drug intervention or physical therapy, thereby promoting the precise management of VVS.

## 5 Conclusion

This study preliminarily proposed and attempted to define the hemodynamic differences between CA-impaired VVS and blood pressure drop-dominant VVS, with preliminary differentiation achieved via multimodal monitoring. This finding deepens knowledge regarding the pathophysiological heterogeneity of VVS and provides novel insights into precise diagnosis, subtype classification, and individualized treatment strategies. This study emphasizes that HUTT combined with TCD, as a multimodal assessment tool, enables synchronous monitoring of cardiac and cerebral hemodynamic parameters, thus facilitating the identification of CA-impaired VVS and enhancing the objectivity and accuracy of test interpretation. Future research should conduct prospective, multicenter, large-sample clinical studies integrated with artificial intelligence to further verify the findings of this study and explore objective diagnostic criteria for defining CA-impaired VVS.

## Data Availability

The original contributions presented in the study are included in the article/supplementary material, further inquiries can be directed to the corresponding author.
